# Mild Myopathy Is Associated with COMP but Not MATN3 Mutations in Mouse Models of Genetic Skeletal Diseases

**DOI:** 10.1371/journal.pone.0082412

**Published:** 2013-11-27

**Authors:** Katarzyna A. Piróg, Yoshihisa Katakura, Aleksandr Mironov, Michael D. Briggs

**Affiliations:** 1 Institute of Genetic Medicine, Newcastle University, Newcastle upon Tyne, United Kingdom; 2 Division of Mechanical Engineering, School of Engineering, Manchester Metropolitan University, Manchester, United Kingdom; 3 Electron Microscopy Core Facility, Faculty of Life Sciences and University of Manchester, Manchester United Kingdom; Weizmann Institute of Science, Israel

## Abstract

Pseudoachondroplasia (PSACH) and multiple epiphyseal dysplasia (MED) are skeletal disorders resulting from mutations in COMP, matrilin-3 or collagen IX and are characterised by short-limbed dwarfism and premature osteoarthritis. Interestingly, recent reports suggest patients can also manifest with muscle weakness. Here we present a detailed analysis of two mouse models of the PSACH/MED disease spectrum; ΔD469 T3-COMP (PSACH) and V194D matrilin-3 (MED). In grip test experiments T3-COMP mice were weaker than wild-type littermates, whereas V194D mice behaved as controls, confirming that short-limbed dwarfism alone does not contribute to PSACH/MED-related muscle weakness. Muscles from T3-COMP mice showed an increase in centronuclear fibers at the myotendinous junction. T3-COMP tendons became more lax in cyclic testing and showed thicker collagen fibers when compared with wild-type tissue; matrilin-3 mutant tissues were indistinguishable from controls. This comprehensive study of the myopathy associated with PSACH/MED mutations enables a better understanding of the disease progression, confirms that it is genotype specific and that the limb weakness originates from muscle and tendon pathology rather than short-limbed dwarfism itself. Since some patients are primarily diagnosed with neuromuscular symptoms, this study will facilitate better awareness of the differential diagnoses that might be associated with the PSACH/MED spectrum and subsequent care of PSACH/MED patients.

## Introduction

Pseudoachondroplasia (PSACH) and multiple epiphyseal dysplasia (MED) are skeletal dysplasias associated with short limbed (upper and lower limbs) dwarfism, lower limb deformities and early onset osteoarthritis [1,2]. They form a distinct spectrum of disease severity and the autosomal dominant forms result from mutations in the genes encoding three extracellular matrix (ECM) molecules, cartilage oligomeric matrix protein (COMP; PSACH and severe MED) [1,3], matrilin-3 (MED) [4] and type IX collagen (MED) [5,6]. PSACH and MED are defined as skeletal dysplasias, or chondrodysplasias, indicating the underlying cartilage pathology. However, patients with these diseases (predominantly those with COMP and type IX collagen gene mutations) are occasionally referred to neuromuscular clinics with symptoms of a ‘non-defined’ mild myopathy before being correctly diagnosed with chondrodysplasia [7-9]. Despite the incorrect referrals and misdiagnoses, little is still known about the effect of these mutant cartilage proteins on the musculoskeletal system as a whole. 

COMP is a large pentameric glycoprotein found in cartilage, ligament, muscle and bone [10,11] and as an extracellular matrix (ECM) bridging molecule it is able to interact with fibronectin [12], aggrecan [13], matrilins [14], collagens [15,16] and integrins [17,18]. PSACH and MED mutations in COMP cluster in two distinct regions of the molecule, namely the thrombospondin type 3 (T3-COMP) repeats and a globular C-terminal domain (CTD-COMP) [7,19]. COMP containing type 3 repeat mutations is partially retained within the ER of chondrocytes from PSACH and MED patients [20] and in various transgenic mouse models, resulting in increased apoptosis and decreased proliferation in the cartilage growth plate [21,22]. However, COMP with the common ΔD469 T3 mutation is secreted in both in the ligaments [15] and tendon[10]. The ECM of mutant cartilage and ligament is also abnormal with more pronounced appearing collagen fibrils in cartilage and a disorganised collagen fibrillar network in the ligament, both of which are suggestive of reduced fibril surface-associated molecules [15,22]. In contrast, COMP containing a variety of CTD mutations can be efficiently secreted into the extracellular matrix (ECM) of cartilage although there may still be mild ER stress in chondrocytes [7,23]. There is also dysregulated apoptosis and decreased proliferation in the growth plates of CTD-COMP mutant mice and the cartilage ECM is abnormal with similarly pronounced collagen fibrils [24]. We have previously shown that COMP is expressed in skeletal muscle, Achilles and patellar tendon and spinal ligament of mice at different ages and that mild myopathy associated with a CTD-COMP mutation is due to an underlying tendinopathy [25] and hypothesised that this pathological feature was most likely due to the role that COMP plays as a catalyst in collagen fibrillogenesis [16,25]. We have previously described clinical data suggesting that myopathy can also be associated with T3-COMP mutations in patients with PSACH and MED [9,26].

Several patients with type IX collagen mutations presenting with MED and mild myopathy have recently been described [8,9]. Moreover, muscle biopsy from a type IX collagen MED patient showed variability in muscle fiber diameters but normal collagen fibers in the perimysial tissue (cross ectional area, fiber distribution and fiber spacing) [27], indicating that type IX collagen mutations may have a direct effect on muscle morphology. 

Matrilin-3 is a tetrameric molecule found predominantly in cartilage and can interact with numerous other ECM molecules including COMP [14,28-30] and type IX collagen [31]. MED mutations cluster predominantly in the β-sheets of the single A-domain of matrilin-3 and result in a canonical ER stress response with dysregulated chondrocyte apoptosis and decreased proliferation in the cartilage growth plate. Denser collagen fibrils in the ECM are also observed when examined by electron microscopy [4,32-34]. Matrilin-3 is thought to be expressed solely in cartilage [29], however, we have previously reported a single MED patient (p.D171..177Edel_insE in matrilin-3) presenting with fatigue during walking which was diagnosed as a neuromuscular condition [26]. This phenotypic presentation suggested a potential influence of mutant matrilin-3 on lower limb weakness, either via underlying muscle/tendon pathology or the indirect result of short-limbed dwarfism on the lower limb strength.

In this paper we present the analysis of three knock-in mouse models of the PSACH/MED disease spectrum, ΔD469 COMP (T3-COMP; PSACH model), T585M COMP (CTD-COMP; mild PSACH model) and V194D matrilin-3 (moderate MED model) [22,34]. We conclude that the PSACH/MED related mild myopathy correlates predominantly with COMP mutations but not a matrilin-3 mutation. This genotype-specific delineation and the detailed analysis of the myopathy and tendinopathy in PSACH and MED mice models will ultimately help in the diagnosis and clinical management of PSACH/MED patients.

## Materials and Methods

### Transgenic mouse models of PSACH and MED

The experimental animal models used in this paper (T585M COMP, ΔD469 COMP and V194D MATN3 mice) were generated as described previously [22,24,34]. As described in these papers the targeting strategy used to generate the knock-in mice was different for the matrilin-3 and the COMP mice. Therefore, appropriate wild type controls of the correct genetic background were generated during standard breeding procedures. Mice homozygous for the relevant mutations were chosen for this study in order to accentuate the muscle and tendon pathology. All experiments were approved by the University Animal Ethical Review Group and performed in compliance with the Scientific Procedures Act of 1986 and the relevant Home Office (under PPL 40/2884) and Institutional regulations governing animal breeding and handling.

### Bone length and grip strength measurement

Wild type and mutant male mice were X-rayed at 3 and 9 weeks of age and Growbase (Certus Technologies) software was used to measure the humeri and the ulnae lengths. Grip strength measurements were performed using a hand-held Chatillon digital force gauge (ChatillonDFE Series Digital Force Gauge, Ametek Inc.) as described previously [25]. Briefly, the mice were preconditioned 20 times on the cage lid then held by the tail and gently lowered towards the apparatus. They were allowed to grip the grid with their forelimbs only and were gently pulled backwards in a horizontal plane. The highest force applied to the grid by the animal (maximum strength) and the force at the moment the grasp was released was recorded. The test was repeated two times per mouse and 10 male mice per genotype were tested at 3 and 9 weeks of age. One-way ANOVA was used for statistical analysis of the data.

### Tissue histology

Full mouse limbs were skinned and fixed in ice-cold 10 % neutral buffered formalin solution (Sigma-Aldrich Ltd; for histology) or in ice-cold 95 % ethanol/5 % acetic acid solution (for immunohistochemistry), decalcified in 20 % (w/v) EDTA pH 7.4 for two weeks, paraffin-embedded and sectioned (6 µm sections). Gomori's trichrome staining (Polysciences Inc.) was performed according to the manufacturer's protocol on transverse limb sections and mounted using a xylene-based mounting solution (Pertex, Surgipath). 3 male mice per genotype were analysed at 3 weeks of age. Muscle fibres with central nuclei were counted and their number was expressed as a percentage of all the muscle fibres seen. One-way ANOVA was used for statistical analysis.

For immunohistochemical analysis, slides with saggital limb sections were dewaxed and rehydrated, endogenous peroxidase activity was quenched in H_2_O_2_/MetOH, followed by antigen unmasking in 0.2 % bovine testes hyaluronidase (Sigma-Aldrich Ltd) in PBS. Samples were blocked in goat serum and 1 % BSA in PBS for 1 h and immediately incubated with primary antibody [COMP (Genetex Inc.), matrilin-3 (R&D), collagen IX (made in-house)] in PBS/BSA for 1 h. Slides were washed in PBS/BSA and incubated with a biotinylated goat anti-rabbit IgG (Dako Cytomation Ltd) in PBS with goat serum, followed by incubation with ABC/HRP reagent (Dako Cytomation Ltd) and developed using DAB chromogen (Dako Cytomation Ltd), with methyl green as counter stain (Vector Labs Ltd). Vectamount™ (Vector Labs Ltd) xylene-free mounting medium was used for mounting the slides.

### Tendon ultrastructure

Wild-type and mutant Achilles tendons were dissected from mice at 3 weeks of age and immediately fixed in 2.5 % glutaraldehyde in 0.1 M sodium cacodylate buffer for 2 h at 4 °C. The tissues were washed three times in 0.1 M sodium cacodylate buffer and fixed in 2 % OsO_4_ in 0.1 M cacodylate buffer for 2 h. They were washed in distilled water and incubated for 2 h in 2 % aqueous uranyl acetate at 4 °C. The tendons were then washed in distilled water, dehydrated in increasing concentrations of acetone (50, 70, 90 and 100 % for 30 min), incubated in propylene oxide to improve resin penetration and 1:1 solution of resin:propylene oxide and embedded in TAAB medium slow resin (TAAB Laboratories Equipment Ltd). Thin 70–80 nm sections were cut with a diamond knife on a Leica ultramicrotome and placed on electron microscope grids. Sections on the grids were stained with silver citrate solution and viewed in a FEI Tecnai 12 Twin transmission electron microscope operated at an accelerating voltage of 80 kV. The fiber diameters were measured, the distribution was analysed for statistical significance using Mann–Whitney *U* test and the differences in the individual fiber frequencies between the animals were assessed using one-way ANOVA.

### Cyclic testing

Cyclic strain test was performed on an Instron 5943 tensile tester (Instron Ltd.). Achilles tendons were harvested at 3 weeks of age and stored in PBS at −80 °C. They were gently thawed and the cross-sectional area was assessed by measuring the thickness in three points along the tendon's length using a microscope graticule (Fisher Scientific). The average mouse tendon length was ^~^5 mm. The tendons were fitted into the tensile tester as shown in [25] using sandpaper strengthened clamps and were kept moist in PBS throughout the experiment. In the cyclic strain test, the starting sample length was 2 mm; the samples were stretched and relaxed with a constant strain amplitude of 0.5 for *n* = 10 cycles (previously established as toe region for the samples [25]) at the strain rate of 0.04/s, and the force and displacement were recorded. The force was normalized against the cross-sectional area, and the decrease in stress at each cycle was compared between the wild-type and mutant samples. Sample stiffness was calculated as F/σ where F=force and σ=displacement. Energy dissipation was calculated as (W_elongation_-W_release_)/(W_elongation_)Independent samples one-way ANOVA was used for statistical analysis.

## Results

### Matrilin-3 is not expressed in mouse muscle or tendon tissues

**Figure 1:**
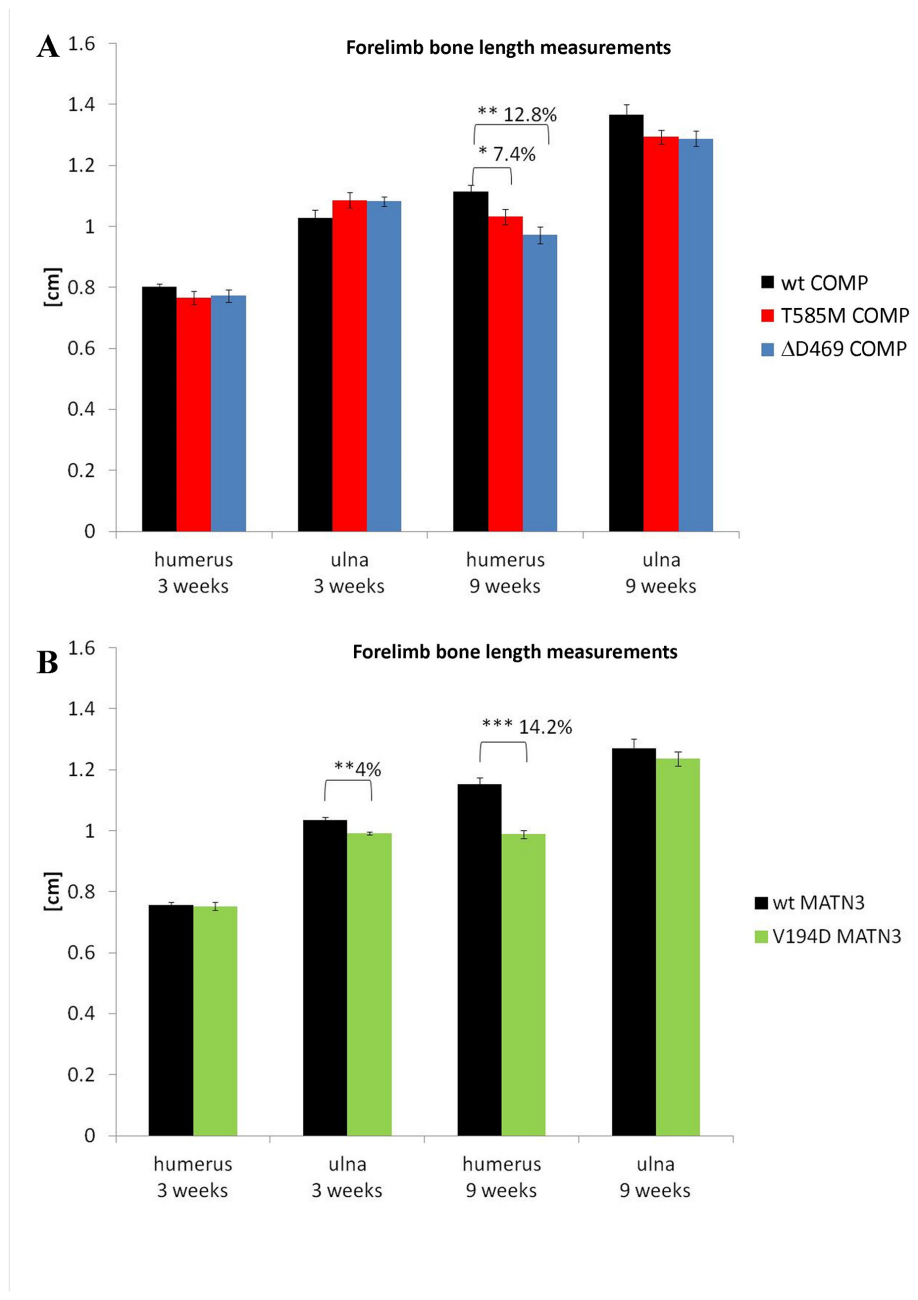
Bone measurements based on X-rays of 3, 6 and 9 week old mice. Humeri in COMP mutant mice (**A**) and in matrilin 3 mutant mice (**B**) were shorter than in the wild type controls. Error bars; SEM (standard error of the mean; n≥10). Key: * P<0.05, ** P<0.01, *** P<0.001.

The localisation of COMP and matrilin-3 in mouse skeletal tissues was analysed using immunohistochemical (IHC) staining performed on saggital sections of 3 week old wild-type hind limbs (Supplemetal Figure 1). COMP protein was detected in the cartilage growth plate (GP), articular cartilage (AC), patellar tendon (PT), meniscus (M), skeletal muscle, Achilles tendon, fibrocartilage of the endo-osseous junction and in the myotendinous junction (Figure S1 and data not shown) [26].We observed no retention of mutant COMP in the skeletal muscle tissue at 3 weeks (not shown). In contrast, matrilin-3 expression appeared be restricted to cartilage and the protein was not detected at appreciative levels in other musculoskeletal tissues (Figure S1).

### COMP and matrilin-3 mutant mice have shorter forelimbs

Bone length measurements were performed at 3 and 9 weeks of age using full skeleton radiographs in order to assess the length of forearms in wild type and mutant mice (Figure 1). Appropriate wild type controls of the correct genetic background were used in all the experiments. The humeri of both COMP mutant mouse lines were shorter than wild-type littermates by 9 weeks of age (Figure 1A; CTD-COMP by 7.4% p<0.05 and T3-COMP by 12.8% p<0.01). There was also a slight trend towards a decrease in the length of the ulnas at 9 weeks of age (p<0.1 and p<0.09 respectively). V194D matrilin-3 mutant mice also had shorter humeri at 9 weeks of age that was in a range similar to the T3-COMP mice (Figure 1B; 14.2% p<0.001). Furthermore, the ulnas were also shorter at 3 weeks of age (by 4% p<0.01) and showed a similar, albeit not statistically significant, trend at 9 weeks of age (p<0.5).

### COMP mutant mice are weaker than their wild type littermates

**Figure 2:**
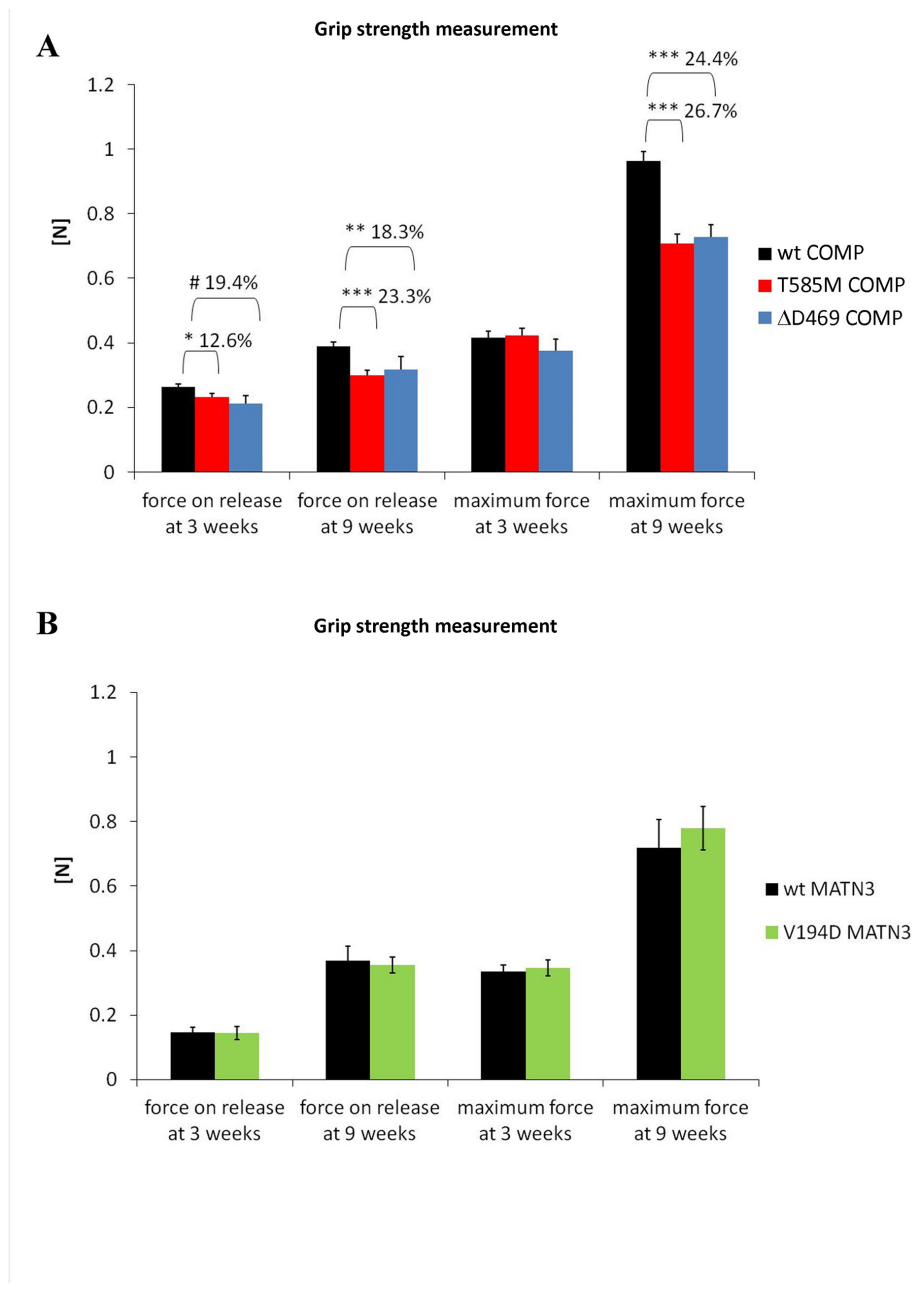
Grip strength measurement at 3 and 9 weeks of age. COMP mutant mice (**A**) become weaker with age compared to their littermates whereas the grip strength of matrilin 3 mutant mice (**B**) remains similar to the wild type littermates at all ages. Error bars; SEM (standard error of the mean; n≥10). Key: * P<0.05, ** P<0.01, *** P<0.001.

 Grip strength measurements were performed as previously described [25] in order to assess wild type and mutant animal muscle strength and stamina at 3 and 9 weeks of age (Figure 2). Mutant mice with the T3-COMP mutation (ΔD469) tired more easily than their wild-type littermates (force on release was 18.3% lower than the wild types, p<0.01) and were generally weaker at 9 weeks of age (maximum force was 24.4% lower than wild type controls; p<0.001, n>10). At 3 weeks there was also a trend similar to that previously described for CTD-COMP (T585M) mice (Figure 2A and [25]). In contrast, despite short-limbed dwarfism, matrilin-3 mutant (V194D) mice did not manifest any muscle weakness with age and all parameters were comparable to wild type littermates at both time points (Figure 2B, n>10). 

### Gomori trichrome staining of gastrocnemius/soleus muscle reveals remodelling events in the muscles of COMP mutant mice

**Figure 3:**
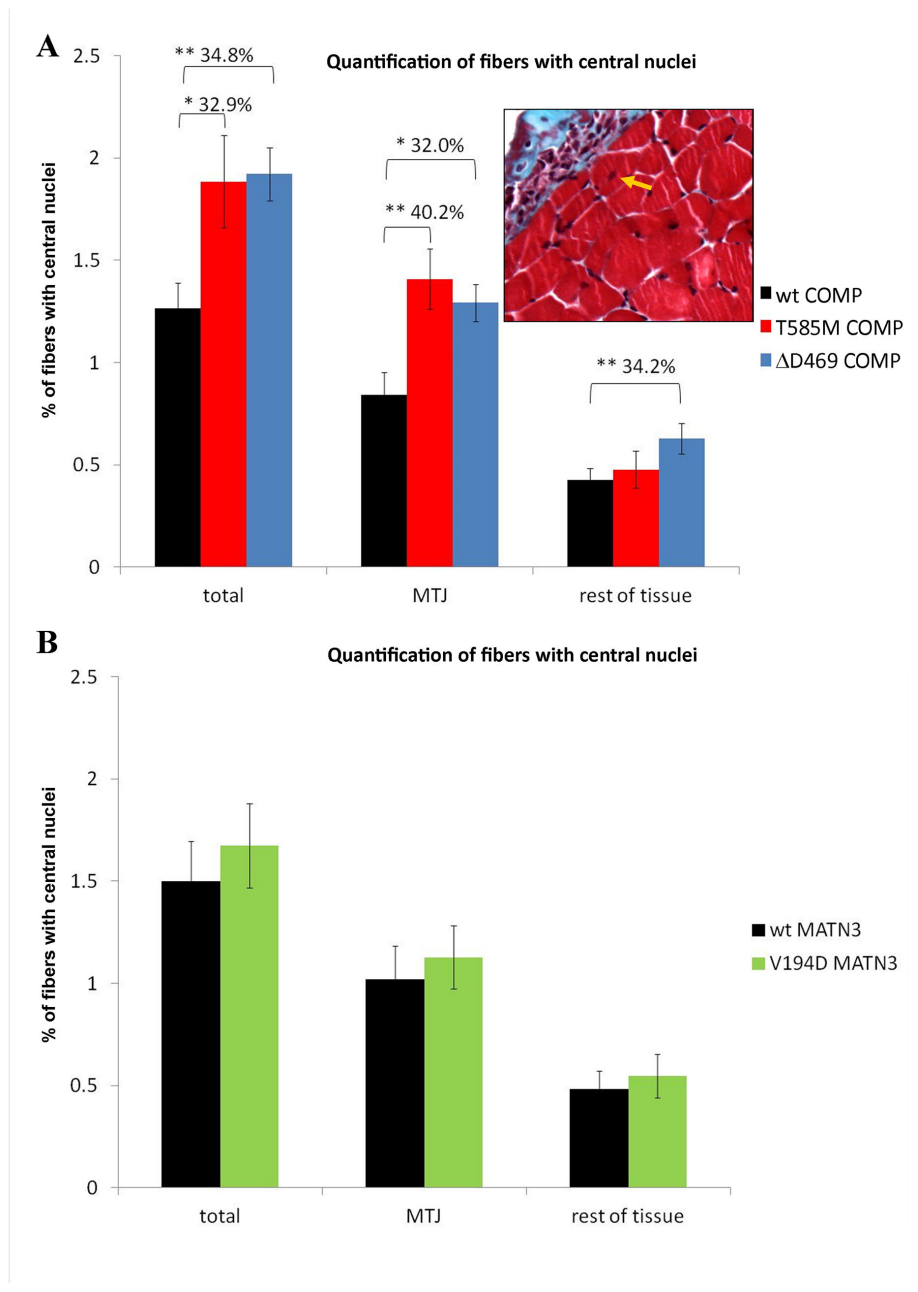
Quantification of the muscle fibers with central nuclei at 3 weeks of age. Quantification based on images obtained using the modified Gomori trichrome stain on transverse sections of mouse legs at 3 weeks of age. Inset shows an example of stained tissue skeletal muscle is stained red, collagenous tissue blue, nuclei black - yellow arrow indicates a muscle fibre with central nucleus. Mutant COMP mice (**A**) have more muscle fibers with central nuclei around the myotendinous and perimysial junction than their wild type littermates. Matrilin 3 mutant mice (**B**) show similar distribution of fibers with central nuclei as their wild type littermates. Error bars; SEM (standard error of the mean; n≥3). Key: * P<0.05, ** P<0.01, *** P<0.001.

 We used modified Gomori trichrome staining to analyse the morphology of the skeletal muscle (gastrocnemius/soleus) in transverse sections of hind limbs from the different mouse models of PSACH and MED. Using this procedure muscle tissue was stained red, cartilage and collagenous tissue (such as tendon and ligament) were stained blue and nuclei were stained black, allowing easy identification of the various tissue components (Figure 3A; inset). This staining protocol was used to analyse the general morphology of the skeletal muscle in the different mouse models and to localise and quantify the muscle fibers with central nuclei, which are indicative of muscle stress and remodelling [35,36]. Since defects to any of the components of the musculoskeletal system may also result in abnormalities to the other tissues [37,38], muscle and tendon from mutant matrilin-3 mice were analysed in detail in order to gain insight into the potential effect of a cartilage and bone growth defect on the morphology and biomechanical properties of the adjoining soft tissues. 

We have found no histological indication of large scale muscle atrophy or deterioration in mutant skeletal muscles. However, T3-COMP mutant muscles contained significantly more fibers with central nuclei at the myotendinous and perimysial junctions (Figure 3A, 32.0% p<0.05), which was similar to the pathology previously reported for CTD-COMP mice (40.2% p<0.01 in [25]). However, there was also a significant increase in the total number of muscle fibers with central nuclei in the rest of the tissue (Figure 3A, 34.2% p<0.01), which was not the case for CTD-COMP [25]. Since COMP is expressed in skeletal muscle as well as in tendon and ligament and the T3-COMP mutations generally have a more severe phenotypic outcome than the CTD-COMP mutations, the T3-COMP mutation may have a more pronounced and measurable effect on the entire skeletal muscle tissue. Mutant matrilin-3 and wild type muscle showed similar numbers and distribution of fibers with central nuclei in all areas analysed (Figure 3B), which further supports our hypothesis that there is no specific muscle pathology in *MATN3*-related MED.

### Ultrastructure analysis of the collagen fibers in Achilles tendons reveals genotype specific changes in tendon morphology

 Achilles tendons from 3 week old wild-type and mutant mice were analysed by transmission electron microscopy (TEM) in order to determine if the different mutations affected the morphology and/or ultrastructure of this tissue. 

**Figure 4:**
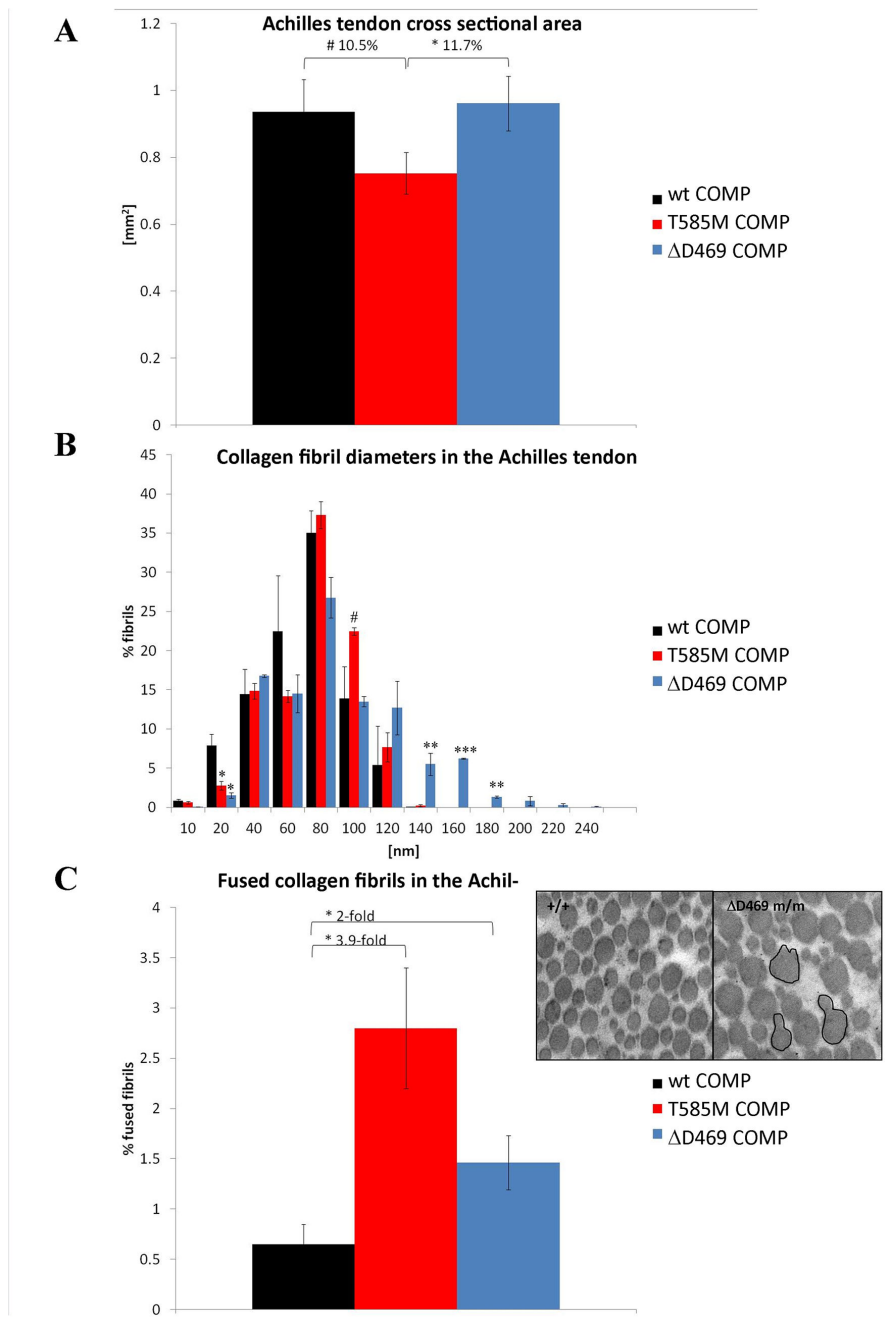
Analysis of ultrastructure of the mutant COMP Achilles tendon at 3 weeks. **A**) Measurement of the cross sectional area of wild type and mutant tendons. T585M COMP tendons but not ΔD469 COMP mutant tendons were thinner than wild type controls at 3 weeks, n>10. **B**) Both COMP mutant tendons contained more of the thicker collagen fibers than the wild type controls, n=3. **C**) Both COMP mutant tendons contained more fused/branching collagen fibers when compared to the wild type tissues at 3 weeks of age, n=3. Error bars; SEM (standard error of the mean). Key: * P<0.05, ** P<0.01, *** P<0.001 (t-test).

As described previously, CTD-COMP mutant mice had thinner tendons as 3 weeks of age (Figure 4A and [25]), however, the cross sectional area of the T3-COMP mutant tendons was comparable to that of wild type samples (Figure 4A). The distribution of fiber diameters in T3-COMP mutant Achilles tendons at 3 weeks of age followed a normal bell curve distribution but broader than the one in wild type mice, with significantly more thicker fibers present in the mutant tendon (Figure 4B). A trend towards a bimodal distribution with a second peak at 160 nm could be seen in the histograms of T3-COMP tendons. A similar distribution was previously found in the CTD-COMP tendons at 9 weeks of age and also in ageing mouse tendons [25,39]. Moreover, the number of fibers per area were comparable between T3-COMP and wild type mice (data not shown) indicating potentially less interterritorial matrix present in the mutant COMP tendons. 

**Figure 5:**
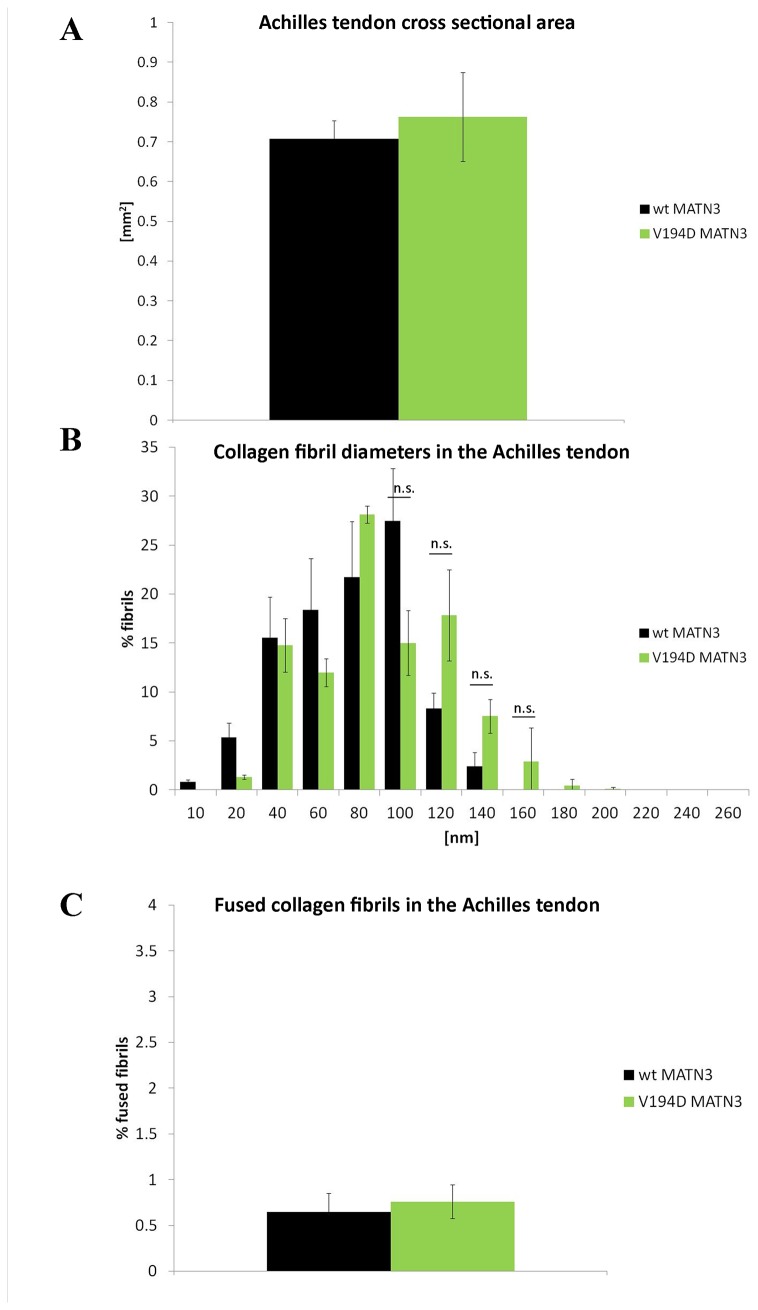
Analysis of the ultrastructure of the mutant MATN3 Achilles tendon at 3 weeks. **A**) Measurement of the cross sectional area of wild type and mutant tendons. V194D MATN3 tendons were not thinner than wild type controls at 3 weeks, n>10. **B**) MATN3 mutant tendons contained similar amounts of both thinner and thicker collagen fibers than the wild type controls, n=3. **C**) MATN3 mutant tendons contained similar amounts of fused/branching collagen fibers when compared to the wild type tissues at 3 weeks of age, n=3. Error bars; SEM (standard error of the mean). Key: * P<0.05, ** P<0.01, *** P<0.001.

Finally, there were more fused/branching collagen fibers (Figure 4C inset) present in T3-COMP mutant Achilles tendons at 3 weeks of age (Figure 4C; ~2-fold p<0.05) and we have previously shown a similar difference for the CTD-COMP mutation (Figure 4C; ~4-fold p<0.05[25]). The trend towards the greater number of bifurcating fibers in the CTD-COMP mutant may be a means for compensating for thinner and therefore more lax tendons. In contrast, mutant matrilin-3 and wild type tendons were the same thickness at 3 weeks of age (Figure 5A) and showed a normal bell curve distribution of collagen fiber diameters (Figure 5B, no statistically significant differences). Furthermore, there was no increase in the number of branching/fused collagen fibers between the wild type and mutant matrilin-3 tendons (Figure 5C).

### Achilles tendons in COMP mutant mice become more lax in biomechanical cyclic testing

**Figure 6:**
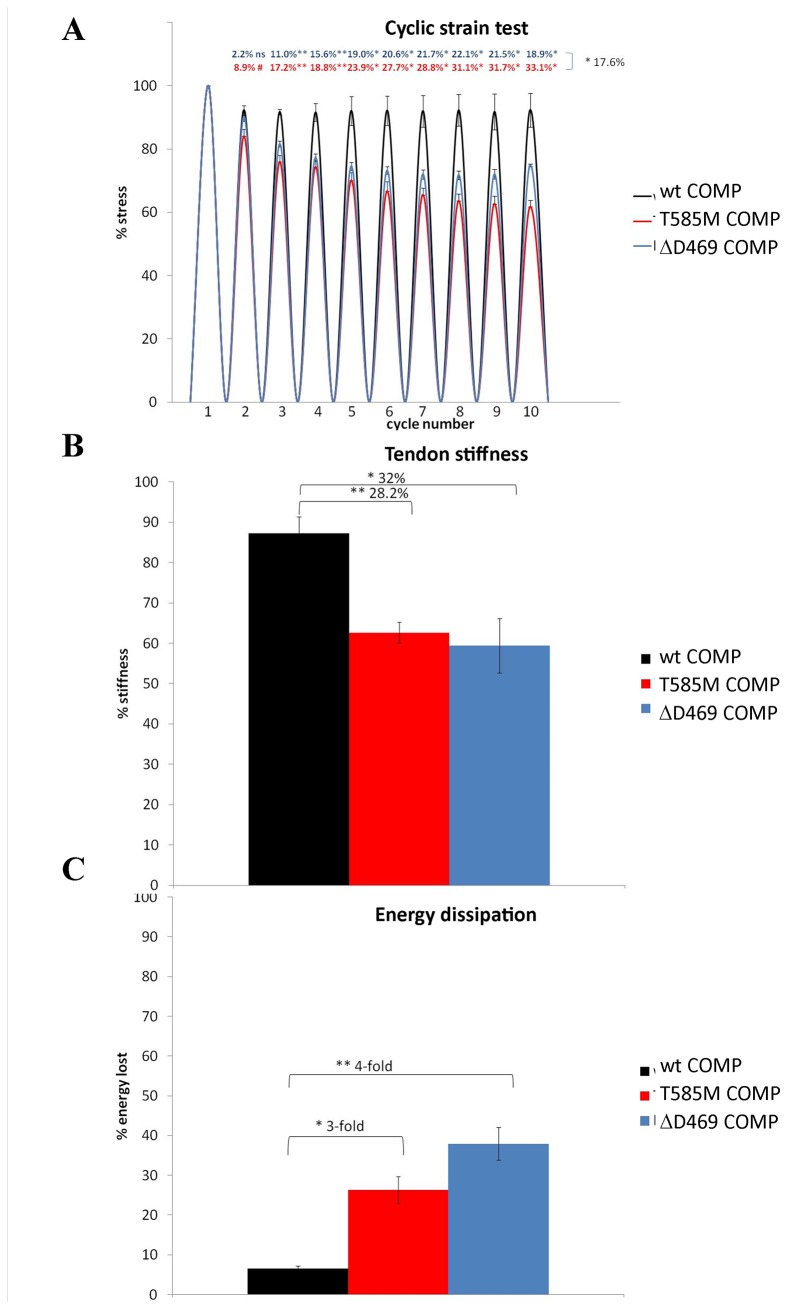
Results of the cyclic test experiment on COMP mutant tendons. **A**) Stress per cycle plot showing a decrease in force needed to extend mutant tendons to the same strain as the wild type controls. Note that although both COMP mutant tendons became more lax in cyclic testing, the ΔD469 COMP tendons become slightly less lax than the T585M COMP tissue by 10^th^ cycle. **B**) Calculation of the proportion of energy dissipated by the Achilles tendons in 10th cycle, showing the mutant COMP tendons may potentially fail as energy storage and buffering elements in mutant hind limbs. **C**) Tendon stiffness at 10^th^ cycle calculated in relation to stiffness at cycle 1 (100%) showing mutant COMP tendons becoming more lax than the wild type controls. Error bars; SEM (standard error of the mean; n≥3). Key: * P<0.05, ** P<0.01, *** P<0.001.

 To assess tendon biomechanical properties, wild type and mutant Achilles tendons from 3 week old mice were used in a cyclic strain experiment as described previously [25]. Tendons were stretched through 10 cycles of a constant strain and the stress at each cycle (measured as force divided by the cross sectional area) was recorded. The tendons from both mutant COMP mice became more lax in cyclic testing, which was indicated by a decrease in both the recorded stress by cycle 10 (Figure 6A) and stiffness (Figure 6B; CTD-COMP 28.2 % p<0.01 and T3-COMP 32 % p<0.05; n≥3). Furthermore, the tendons from CTD-COMP mutant mice were significantly more lax than tendons from T3-COMP mutant mice by cycle 10 (Figure 6A; 17.6 %, p>0.05). Normal energy dissipation for an animal tendon varies between 8-18 % upon release [40,41]. Energy dissipation values calculated for COMP wild type tendons fall within this range. COMP mutant tendons were dissipating more energy than the wild type tissue as the cycling progressed indicating they were failing in their energy storage and muscle buffer roles (Figure 6C; CTD-COMP 3-fold p<0.05, T3-COMP 4-fold p<0.01; n≥3). 

**Figure 7:**
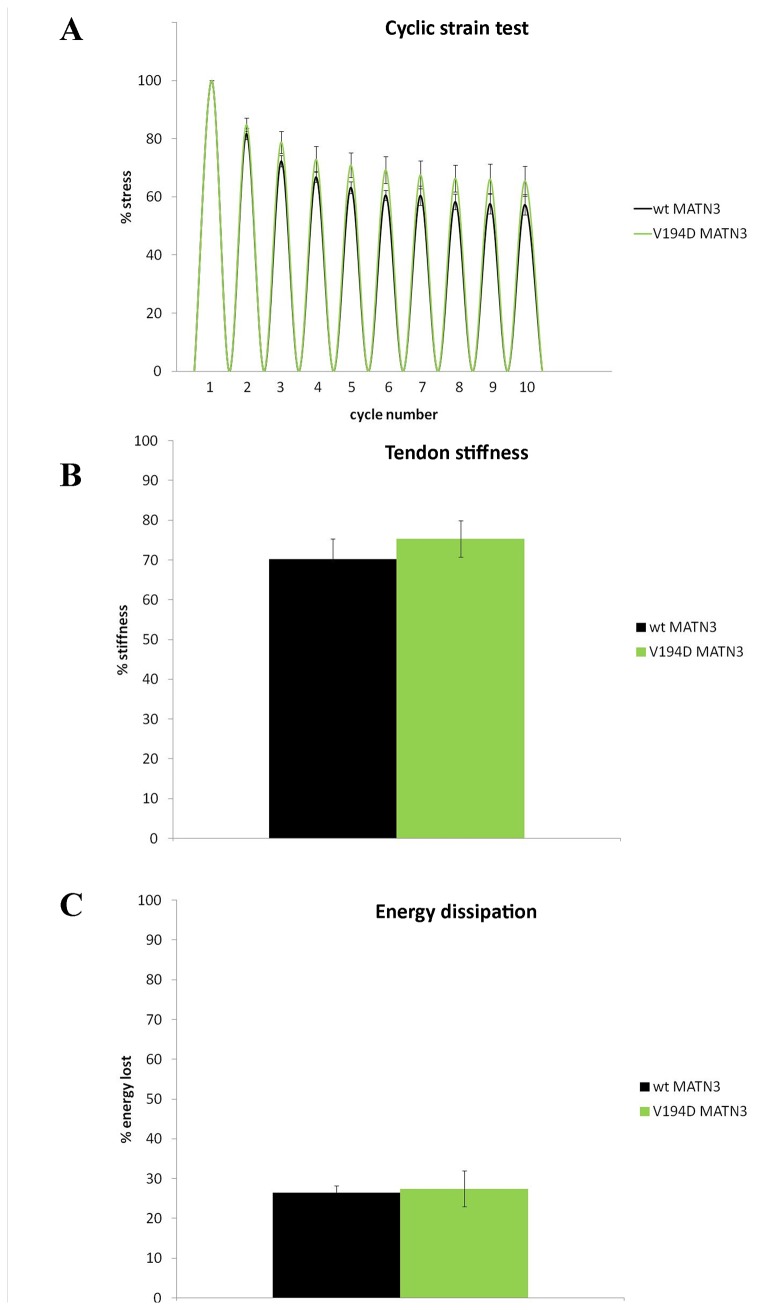
Results of the cyclic test experiment on matrilin 3 mutant tendons. **A**) Stress per cycle plot showing a decrease in force needed to extend mutant tendons to the same strain as the wild type controls. We found no difference between the wild type and mutant tendons at 3 weeks. **B**) Calculation of the proportion of energy dissipated by the Achilles tendons in 10th cycle, showing no difference between the wild type and mutant matrilin 3 tissue at 3 weeks. **C**) Tendon stiffness at 10^th^ cycle calculated in relation to stiffness at cycle 1 (100%) showing mutant matrilin 3 tendons did not become more lax than the wild type controls in the cyclic testing experiment (n≥3).

Achilles tendons from mutant matrilin-3 mice behaved in a similar way to wild type tendons in cyclic testing and the maximum stress, energy loss and stiffness recorded at cycle 10 was comparable to wild type controls (Figure 7A-C), suggesting that the matrilin-3 mutation has no direct effect on the biomechanical properties of Achilles tendon in mice.

## Discussion

Pseudoachondroplasia (PSACH) and multiple epiphyseal dysplasia (MED) are skeletal dysplasias associated with short-limbed dwarfism and early onset osteoarthritis (OA). However, recent clinical findings suggest that there is also a neuromuscular component associated with these diseases that may actually manifest prior to the skeletal phenotype. Furthermore, soft tissue abnormalities such as ligamentous laxity, myopathy and joint instability may cause, or exacerbate, the lower limb deformities (such as *genu varum* and *genu valgum*) and may further aggravate the early onset osteoarthritis seen in PSACH and MED patients [42,43]. Therefore, a thorough characterisation of the tendomuscular pathology associated with PSACH and MED would enable better differential diagnosis and clinical management of affected individuals.

Patients with PSACH/MED and CTD-COMP or type IX collagen mutations presenting with ‘neuromuscular-like’ symptoms prior to a correct diagnosis of skeletal dysplasia have been described on several occasions [7-9,26,27,44]. Based on these reports we have previously analysed a CTD-COMP (T585M) model of mild PSACH and demonstrated that muscle weakness in this mouse was due to an underlying tendinopathy [25]. As a result of recent findings describing neuromuscular-like symptoms in T3-*COMP* and possibly *MATN3* patients [9,26], we wished to characterise the muscle and tendon phenotype in two further mouse models of the PSACH/MED spectrum; the *Comp* ΔD469 [22] and *Matn3* V194D [34] models of severe PSACH and moderate MED respectively.

Cartilage oligomeric matrix protein (COMP) is a large pentameric glycoprotein expressed in skeletal muscle and tendon/ligament as well as in cartilage [10,11,25]. Type IX collagen is found in cartilage [45,46], fibrocartilage [47] and vitreous of the eye [45], whilst some reports also describe the mRNA of at least one of the type IX collagen chains in skeletal muscle [27,48]. The expression of matrilin-3 has only been reported in cartilage to date [29,49,50]. We therefore decided to confirm the expression of these molecules in mouse tissues before proceeding with further biological experiments. Using immunohistochemistry and real time PCR [25] we have confirmed that COMP is expressed in skeletal muscle, myotendinous junction, fibrocartilage, Achilles and patellar tendon in the hind limb and spinal ligament at 3 weeks of age, as well the cartilaginous growth plate. We were also able to show that type IX collagen is present in cartilage, fibrocartilage at the tendo-osseous junction and to a much lesser extent in the skeletal muscle (not shown). In contrast, matrilin-3 was only detectable in cartilage thus confirming its restricted distribution. 

We measured the bone lengths of forelimbs in our experimental models prior to mechanical testing and determined that short-limbed dwarfism was within a similar range for all the models tested. We then performed grip test measurements and found that T3-COMP mutant mice were weaker at 9 weeks of age and tired more easily than their wild type littermates, thus confirming a similar trend in progressive muscle weakness as previously published for CTD-COMP mice [25]. However, in contrast, V194D matrilin-3 mice did not show any signs of muscle weakness or fatigue at 3 or 9 weeks of age, thus proving that the muscle weakness observed in patients of the PSACH/MED disease spectrum is not the result of short limbed dwarfism alone.

We next performed a histological examination of the skeletal muscle morphology in our mouse models and counted the incidence of muscle fibers with central nuclei. Such fibers are common in foetal muscles [51] and in adult muscle are an indicator of myofiber stress and remodelling [35,36]. We found an increase in the amount of fibers with central nuclei at the myotendinous and perimysial junctions in both COMP mutant models at 3 weeks of age, indicating that the COMP-related myopathy is potentially due to an abnormality in the collagenous connective tissues surrounding the muscle itself. The morphology of skeletal muscle in the matrilin-3 mutant mice was normal, thus confirming that the shorter bones in the matrilin-3 mutant mice do not affect muscle regeneration and remodelling.

Ultrastructural studies of the Achilles tendons at 3 weeks of age were performed to assess the effect of mutant proteins on tendon morphology. We found that although the collagen fiber diameters in both COMP mutant tendons did follow the normal distribution, CTD-COMP and T3-COMP mutant tendons both contained thicker collagen fibers when compared to the wild type controls, further confirming our previously reported observations for the CTD-COMP mutant mice [25]. The total cross sectional area of T3-COMP mutant tendons was similar to the wild type tissues, unlike the thinner tendons in the CTD-COMP mutant mice [25]. The fibers per area count was comparable for T3-COMP and wild type mice which in conjunction with the finding of thicker collagen fibers in the mutant tendon indicates potentially less interterritorial matrix present in the ΔD469 mutant COMP tendons. Proteoglycans in the tendon ECM are important for water retention and lubrication of the tissue and may therefore influence its biomechanical properties [52]. Previous studies of a transgenic ΔD469 COMP mouse model showed a partial loss of proteoglycans and aggrecan in the interterritorial matrix of growth plate cartilage [53]. It is therefore tempting to speculate that the T3-COMP mutation would have a similar effect on the interterritorial matrix in the Achilles tendon. 

By contrast, the fibers per area count was lower for CTD-COMP mice than for the wild type controls and the amount of extracellular matrix proteoglycans appeared comparable to the wild type controls (by DMMB staining when normalised to DNA content [25]). CTD-COMP tendons were also significantly thinner than the wild type tissues, indicating that a CTD-COMP mutation may have a more profound effect on ECM composition and its ability to swell and retain water during the measurement of tendon diameters in PBS. Furthermore, it is known that intermittent high loads (such as running) may result in a larger tendon cross-sectional area. Since CTD-COMP mice were the only mouse model where early onset OA was documented [24], they may have also been less active; this may in turn have resulted in thinner Achilles tendons. However, this aspect of PSACH/MED tendon biology remains to be clarified. 

T3-COMP mutant tendons contained more fused/bifurcating collagen fibers than the wild type tissues, again recapitulating the phenotype seen in CTD-COMP mice and possibly indicative of microdamage/remodelling in the tendon. Such a feature is normally observed in foetal tendon and in adult tendon scar tissue [54,55]. It is thought that bridges between the collagen fibers may be an attempt to strengthen the biomechanical properties of the tissue, and in the case of COMP-related tendinopathies it may be a way to compensate for the increased tissue laxity. Similar bifurcating collagen fibers have also been reported in PSACH patients [15,25]. In contrast, no structural abnormalities were found in the matrilin-3 mutant tendons thus confirming again that short limbed dwarfism *per se* does not have an effect on the musculoskeletal system.

We tested the biomechanical properties of wild type and mutant tissues using the cyclic strain test at 3 weeks of age [25]. We found that the T3-COMP mutant Achilles tendons became more lax with cyclic testing, similar to what we have previously shown for CTD-COMP mutants [25] and again repeated in this study. Moreover, changes in tissue stiffness calculated for the first and last cycle pointed to increased tendon laxity in COMP mutant tendons. However, it seemed that the ΔD469 COMP tendons were slightly more resilient to stretching than the T585M COMP tissue, especially by the last cycle, which could be due to the larger cross sectional area and the fiber per area count of the T3-COMP mutant tendon compared to the CTD-COMP tissue. The observed laxity of the mutant tendon under stretch conditions possibly recapitulates the joint laxity seen in COMP-associated PSACH/MED patients which in turn may potentially exacerbate disease complications such as early onset OA [56]. In addition, COMP mutant tendons dissipated more energy during cyclic strain testing than the wild type controls, indicating that their energy storage potential and the role of buffer for the skeletal muscle were somewhat impaired during the test. This may in turn explain why the pathology in the skeletal muscle is seen predominantly at the myotendinous junction and perimysial junctions, which are the collagenous fibrous tissues important for conveying forces between tendon and the muscle [52,57,58]. In contrast, matrilin-3 mutant Achilles tendons behaved similar to wild type tissues in the cyclic test and did not become more lax than the controls. This is in correlation with the disease profile of the majority of matrilin-3 MED patients, whereby only skeletal development is affected and there are no soft tissue complications [56]. We can only surmise that the matrilin-3 patient previously reported in our cohort (D171..177Edel_insE) carries an unrelated mutation leading to the muscle weakness phenotype, or alternatively, since matrilin-3 is also expressed in the calcaneous perhaps such a large deletion may lead to a gross protein malfunction and further tissue instabilities at this attachment point.

Overall, our data suggest that PSACH/MED related muscle weakness correlates with mutations in COMP but not in matrilin-3. This is in agreement with the spatial distribution of these two proteins in the mouse hind limb and the role these proteins play in the musculoskeletal tissues. COMP is a know catalyst of collagen fibrillogenesis [16] and is present in the interterritorial matrix in tendon [11]. Studies in horses have shown it to be an important factor in tendon growth [59]. Furthermore, CTD-COMP mutations have been shown previously to lead to thicker amorphous collagen fibers *in vitro* [60] and both CTD- and T3-COMP mutant mice show differences in the ultrastructure of extracellular matrix in the growth plate cartilage [22,24]. It is therefore plausible to hypothesise that mutations in COMP affect collagen fibrillogenesis in mutant tendons as well. This may then lead to changes in the biomechanical properties of the tissue, joint laxity, insufficient energy storage, force distribution and buffering during walking and other motor activities leading in turn to the observed lower limb weakness. Our data presented in this paper supports this hypothesis. Furthermore, it has been suggested that ageing may enhance MMP-related degeneration of the ECM in cyclically strained tendon, which can be measured by the release of COMP fragments and subsequent loss of tensile strength [61]. It is therefore interesting to speculate this process could be further exacerbated in the case of PSACH/MED structurally abnormal tendons. 

Type IX collagen related MED with a myopathy has been reported previously [8,27]. A biopsy of skeletal muscle of an MED patient with COL9A3 mutation showed variability in muscle fiber diameters with normal perimysial morphology [27] indicating that mutant type IX collagen may have a direct effect on the skeletal muscle morphology and function. However, a mouse model is currently not available for analysis and the musculoskeletal complications related to type IX collagen mutations remain to be investigated further.

## Conclusions

To conclude, the tendomuscular complications in PSACH/MED patients are often diagnosed based on changes in muscle strength,. This detailed analysis of the muscle and tendon phenotype in PSACH/MED mouse models has revealed that pathological changes in the skeletal muscle are located in certain specific areas (in particular the myotendinous and perimysial junction) and stem from an underlying tendon defect. The severe end of the PSACH/MED disease spectrum is caused exclusively by the mutations in COMP, the molecule with the broadest tissue distribution and direct involvement in collagen fibrillogenesis. It is therefore interesting to speculate that if indeed the muscle weakness and tendon/ligament laxity have an effect on subsequent disease severity and complications (such as the severity of the observed osteoarthritis or lower limb deformities), perhaps some of those features could be alleviated with a tailored physiotherapy regime and better patient management in the future [62-65]. 

## Supporting Information

Figure S1 **Localisation of COMP and matrilin 3 in musculoskeletal tissues.** **A**) in knee joint, **B**) skeletal muscle (immunohistochemistry, brown staining). Negative control was generated following the standard staining procedure with no primary antibody. Scale bar 200μm. Key: GP-growth plate cartilage, AC-articular cartilage, M-meniscus, PT-patellar tendon.
(TIF)
