# Supplementary figures and images for: Mild Myopathy Is Associated with COMP but Not MATN3 Mutations in Mouse Models of Genetic Skeletal Diseases
Source: PLoS One. 2013 Nov 27;8(11):e82412. doi: 10.1371/journal.pone.0082412 (PMC3842254; doi:10.1371/journal.pone.0082412)

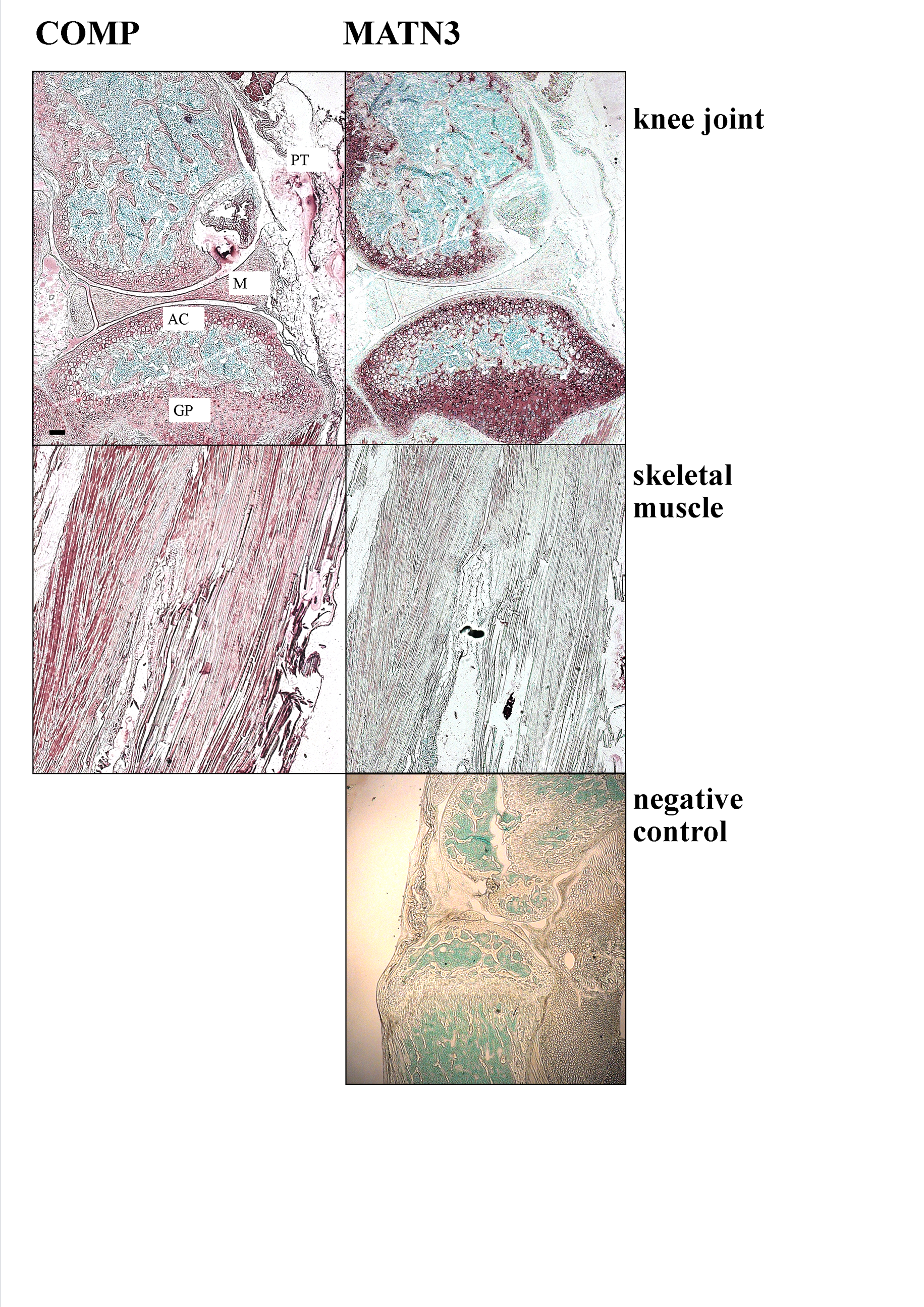

Supplement: Figure S1 — Localisation of COMP and matrilin 3 in musculoskeletal tissues. A) in knee joint, B) skeletal muscle (immunohistochemistry, brown staining). Negative control was generated following the standard staining procedure with no primary antibody. Scale bar 200μm. Key: GP-growth plate cartilage, AC-articular cartilage, M-meniscus, PT-patellar tendon. (TIF) [file pone.0082412.s001.tif]
